# Integration of Mindfulness and Acupuncture After Spine Surgery: Protocol for a Randomized Acceptability and Feasibility Trial

**DOI:** 10.2196/77236

**Published:** 2025-11-17

**Authors:** Trevor A Lentz, Laura S Porter, Lewis McGarvey, Cassandra Rhodes, Brett Rocos, Tina Gremore, Emily K Reinke, Stephanie A Eucker, Cynthia L Green, Emily Poehlein, Austin Dixon, Michelle Mill, Suzanne Finley, Christine M Goertz

**Affiliations:** 1 Department of Orthopaedic Surgery, Duke University, 311 Trent Drive, Durham, NC, 27710, United States, 1 9196687495; 2 Duke Clinical Research Institute, Duke University, Durham, NC, United States; 3 Robert J. Margolis Institute for Health Policy, Duke University, Durham, NC, United States; 4 Department of Psychiatry and Behavioral Sciences, Duke University, Durham, United States; 5 Department of Emergency Medicine, Duke University, Durham, United States; 6 Department of Biostatistics and Bioinformatics, Duke University, Durham, NC, United States; 7 Practice Space, Durham, NC, United States; 8 Blue Sky Acupuncture, Chapel Hill, NC, United States

**Keywords:** integrative, complementary, pain, chronic pain, opioids

## Abstract

**Background:**

Spine surgery is increasingly common in the United States, contributing substantially to spine-related health care costs. While many patients benefit, up to 25% experience chronic postsurgical pain, and the procedure is linked to high rates of opioid misuse. Key risk factors for persistent pain and opioid use include poorly controlled early postsurgical pain, high pain-sensitivity, and poor pain-coping. Clinical guidelines recommend multimodal treatment to address these risks, but such approaches are not well studied or widely implemented. Combining two safe and effective nonpharmacologic treatments, specifically mindfulness and acupuncture that target these factors, has the potential to improve postsurgical recovery and reduce the incidence of chronic pain and opioid use.

**Objective:**

This paper describes the study protocol for the Integrating Mindfulness and Acupuncture after Spine Surgery (I-MASS), which is a single-site, 2-arm randomized controlled trial that will assess the feasibility and acceptability of a novel multicomponent program integrating mindfulness delivered via a mobile app, acupuncture, and education for patients undergoing single-level spine surgery.

**Methods:**

A total of 50 participants will be randomized 1:1 to receive (1) mindfulness and acupuncture (ie, I-MASS program) plus enhanced education or (2) enhanced education alone. Mindfulness training will consist of a 4-week app-based program starting 1 week prior to surgery, and acupuncture will include up to 8 visits (1 visit prior to surgery and 7 after surgery) during the 13-week program. Enhanced education appropriate for each phase of recovery will be delivered through the mobile app. Primary outcomes are recruitment eligibility and enrollment rates, mindfulness module and acupuncture visit completion rates, questionnaire completion rates, and participant satisfaction. Secondary outcomes include physical function, fatigue, pain interference, depressive symptoms, anxiety, ability to participate in social roles and activities, sleep disturbance, fear avoidance beliefs, pain intensity, pain medication use, adverse events, hospital readmissions, and emergency department visits.

**Results:**

Trial enrollment began in August 2024. As of May 9, 2025, 35 participants have been enrolled. Data analysis has not yet been performed. Enrollment is expected to be completed in the fall of 2025.

**Conclusions:**

The I-MASS program addresses the need for mind and body treatments to improve recovery and reduce the risk of persistent pain and opioid use after spine surgery. This integrated model of care is designed to be user-friendly and scalable, enhancing its potential for implementation in real-world settings. A future pragmatic trial is planned to determine if the I-MASS program results in better outcomes compared to either treatment alone or usual care.

## Introduction

Spine-related pain, encompassing low back and neck pain, is a leading cause of disability and a major driver of health care expenditures in the United States [1,2]. Many patients undergo spine surgery to alleviate pain; however, up to 25% of patients continue to experience chronic postsurgical pain, defined as pain persisting for at least 3 months after surgery [3,4]. Chronic postsurgical pain not only exacerbates disability and diminishes quality of life but also increases the risk of prolonged opioid use, additional invasive procedures, and associated complications [5-8]. The management of chronic postsurgical pain is heavily dependent on opioids, which often fail to provide adequate pain relief [9,10] and carry serious risks related to misuse, addiction, and overdose [11]. Patients undergoing spine surgery are at high risk of long-term opioid use, with the cumulative dose of opioids prescribed in the immediate postoperative period being a strong predictor of prolonged use [12,13].

Chronic postsurgical pain develops through complex physiological and psychosocial mechanisms [14-18]. As a result, guidelines for postsurgical pain management advocate for a comprehensive, multimodal approach that targets modifiable risks for chronic pain [18,19], including depression, anxiety, and maladaptive pain-related beliefs like catastrophizing [20-23]. Multimodal treatment combines various analgesic medications and nonpharmacologic interventions to optimize pain relief [19,24]. Nonpharmacologic treatments, including mindfulness and cognitive behavioral therapies, are recommended to enhance pain coping mechanisms and reduce pain-related anxiety [25]. Despite these guidelines, multimodal care, especially the use of nonpharmacologic treatments, remains underused in clinical practice.

The Integrating Mindfulness and Acupuncture after Spine Surgery (I-MASS) study aims to address this gap by developing and assessing the feasibility of a novel program that integrates mindfulness, acupuncture, and education for patients undergoing spine surgery. Mindfulness is a psychological intervention that enhances coping and reduces pain-related anxiety, avoidance, and catastrophizing [26-28]. It involves techniques such as meditation, body awareness, and exploration of patterns of behavior, thinking, feeling, and action [29]. These techniques help moderate the influence of pain on stress, resulting in less pain-related stress arousal and a reduced desire for opioids [30,31]. Mindfulness also fosters long-term self-management and coping skills, contributing to improved emotional regulation and relaxation [32]. Acupuncture involves the insertion of thin needles into specific anatomical points on the body. The technique is used to alleviate pain, treat various health conditions, and promote overall well-being. Acupuncture provides physical therapeutic inputs that induce pain relief through neurochemical changes, such as endorphin production and autonomic mechanisms [33,34]. It reduces neuroinflammation and slows the development of chronic sensitization and pain through effects on endogenous opioid and inflammatory pathways [35-38].

Combining mindfulness and acupuncture offers a promising approach to preventing chronic postsurgical pain, reducing opioid use, and enhancing recovery following spine surgery. The distinct yet complementary mechanisms through which these interventions operate are hypothesized to yield additive and potentially synergistic effects [39], addressing both biological and psychological risk factors for the development of chronic pain [18,30,32-34,40,41]. While mindfulness helps patients respond more effectively to pain and develop long-term coping skills, acupuncture can provide short-term analgesia and reduce pain sensitivity [34]. Both treatments also share common mechanisms such as modulation of the autonomic nervous system and improvement of emotional regulation, further enhancing their combined efficacy [30,35]. Very few studies have fully integrated these treatments and aimed to assess their combined effects on pain and recovery following spine surgery [39].

This integrated model of care is designed to be user-friendly and scalable. In I-MASS, mindfulness training is delivered through a mobile app platform, enhancing its potential for implementation in real-world settings and providing an attractive option for postoperative pain management. Although highly promising, a rigorous, pragmatic trial is needed to determine if the I-MASS program results in lower rates of chronic pain, pain-related disability, and opioid use compared to either treatment alone or as an alternative to usual care following spine surgery. Trials of this size and scope require careful preparation, which includes feasibility testing of the novel intervention and research protocols, as outlined in the National Institutes of Health (NIH) Stage Model [42].

This paper describes the study protocol for a single-site, 2-arm randomized feasibility and acceptability trial of mindfulness and acupuncture (ie, the I-MASS program) plus enhanced patient education compared to enhanced patient education alone in patients undergoing spine surgery. Outcomes will focus on the feasibility and acceptability of I-MASS, the feasibility of recruitment and retention strategies, and data collection procedures through both the mobile app platform and electronic health record (EHR). Feasibility will be assessed through mindfulness module completion rates, acupuncture treatments attended, participant retention, and questionnaire completion rates. Acceptability will be assessed by patient-reported satisfaction, acceptability, and usability measures.

## Methods

### Study Design

This study uses a single-site, 2-arm, randomized controlled trial design to assess the feasibility and acceptability of the I-MASS program. The intervention arm will consist of the I-MASS program plus enhanced education, while the comparison arm will consist of enhanced education alone. Individual participants will be randomized 1:1 without stratification into each study arm. A target sample size of 50 participants has been established to determine the feasibility and acceptability of the protocol.

### Setting

All participants in the study will be recruited from the Duke University Health System (DUHS). DUHS is based in Durham, North Carolina, and includes 3 hospitals and over 140 primary and specialty care clinics across central and eastern North Carolina. In the fiscal year 2023, DUHS served nearly 67,000 inpatient stays and 5 million outpatient visits. Acupuncture will be provided by licensed, study-approved acupuncturists at clinics in the surrounding geographic region, and patients will be free to choose their preferred site.

All mindfulness interventions, delivery of enhanced education in both arms, and collection of patient-reported outcomes in both arms will occur in a mobile app platform administered by Pattern Health, a software company based in Durham, North Carolina. Pattern Health provides an online dashboard for study teams to track all trial participants’ activity in the app, including completion of questionnaires and access to educational and mindfulness content. The dashboard permits easy export of participant use data for analysis.

### Data and Safety Monitoring

An independent monitoring committee (IMC) oversees this trial. All members are independent of the investigators involved in conducting the trial. IMC responsibilities include reviewing biannual reports to (1) ensure participant safety and (2) advise the principal investigators regarding scientific and ethical conduct. The IMC evaluates adverse event data, protocol deviations, reasons for exclusion, and participant accrual. IMC members make recommendations to the principal investigators and the funding agency regarding continuation, termination, or other modifications of the randomized controlled trial.

### Regulatory Approvals

The trial protocol received ethical approval from the Duke University institutional review board (IRB; Pro00114814). The trial protocol was also approved by the National Center for Complementary and Integrative Health at NIH. All investigators and clinicians, including study acupuncturists outside the DUHS, completed training on the protection of human subjects.

### Eligibility Criteria

To be included in the study, patients must meet all the following criteria: (1) be undergoing single-level fusion, discectomy, or laminectomy of the cervical or lumbar spine for pain management at DUHS; (2) be aged ≥18 years; and (3) have access to a smartphone or mobile device (with an Android or iOS operating system) and internet to complete training and questionnaires. The scope and complexity of spine procedures were limited to increase homogeneity within the cohort and to reduce potential confounding variables for this feasibility and acceptability study.

Patients will be excluded if they meet any of the following criteria: (1) having conditions that make consent, follow-up data collection, or use of the intervention prohibitive (eg, non-English speaking, serious psychiatric conditions such as schizophrenia, traumatic brain injury, or dementia-type illness); (2) undergoing surgery for neoplastic disease; (3) current daily opioid use greater than 100 mg morphine equivalents; (4) unable to receive acupuncture due to injury, infection, or other contraindication to the use of needles at acupuncture sites; (5) are unable to attend outpatient clinic for acupuncture follow-up (eg, from out-of-state); (6) currently practicing mindfulness or receiving acupuncture; or (7) being in hospice or receiving palliative care. Exclusion criteria are assessed using an initial self-report screen, and study personnel perform a medical record review to confirm criteria that can be assessed through the medical record (eg, prior diagnoses, opioid prescriptions, neoplastic disease, and planned surgery).

### Identification, Recruitment, and Enrollment

We will use the following three strategies to identify and recruit participants:

1. Recruitment through Maestrocare: our primary form of recruitment will be through Maestrocare, Duke University’s version of the Epic electronic health record system. A weekly customized report will be generated in Epic to identify potential participants based on searchable eligibility criteria. Automated messages will be sent through MyChart to patients meeting these criteria and scheduled for surgery in 21 to 42 days (3‐6 wk). This timeframe allows adequate time to consent, randomize, and deliver preoperative interventions. The automated message informs them of their potential eligibility for the study and includes a link to alert our research coordinators of their initial interest. A reminder message will be sent after 5 days if the patient has not responded to the initial message. Patients who indicate interest will be prompted to complete a prescreen questionnaire in REDCap (Vanderbilt University) [43,44] to confirm eligibility. Those who meet the initial eligibility criteria will be contacted by phone by a research coordinator. All patient contacts will be recorded in a screening log, along with the outcome of the call.
2. Recruitment by phone: for patients who do not respond to the MyChart message, the research coordinator will contact them by phone, conduct screening for study participation, provide additional information on the study, and answer any questions.
3. Recruitment by clinic: patients may also be recruited in the clinic after they have decided to undergo surgery. Patients will be introduced to the study by their surgeon or another member of their health care team. The introduction will include basic study information, which may consist of distributing an IRB-approved study flyer, and patients will be asked if they are interested in learning more about the study. Those interested in learning more will be directed to the study flyer QR code that links to the REDCap screening form. The clinician may also contact the study coordinator with patient contact information if permitted by the patient. Those meeting the initial criteria will be contacted by phone by a research coordinator.

Recruitment through Maestrocare: our primary form of recruitment will be through Maestrocare, Duke University’s version of the Epic electronic health record system. A weekly customized report will be generated in Epic to identify potential participants based on searchable eligibility criteria. Automated messages will be sent through MyChart to patients meeting these criteria and scheduled for surgery in 21 to 42 days (3‐6 wk). This timeframe allows adequate time to consent, randomize, and deliver preoperative interventions. The automated message informs them of their potential eligibility for the study and includes a link to alert our research coordinators of their initial interest. A reminder message will be sent after 5 days if the patient has not responded to the initial message. Patients who indicate interest will be prompted to complete a prescreen questionnaire in REDCap (Vanderbilt University) [43,44] to confirm eligibility. Those who meet the initial eligibility criteria will be contacted by phone by a research coordinator. All patient contacts will be recorded in a screening log, along with the outcome of the call.

**Figure 1.**
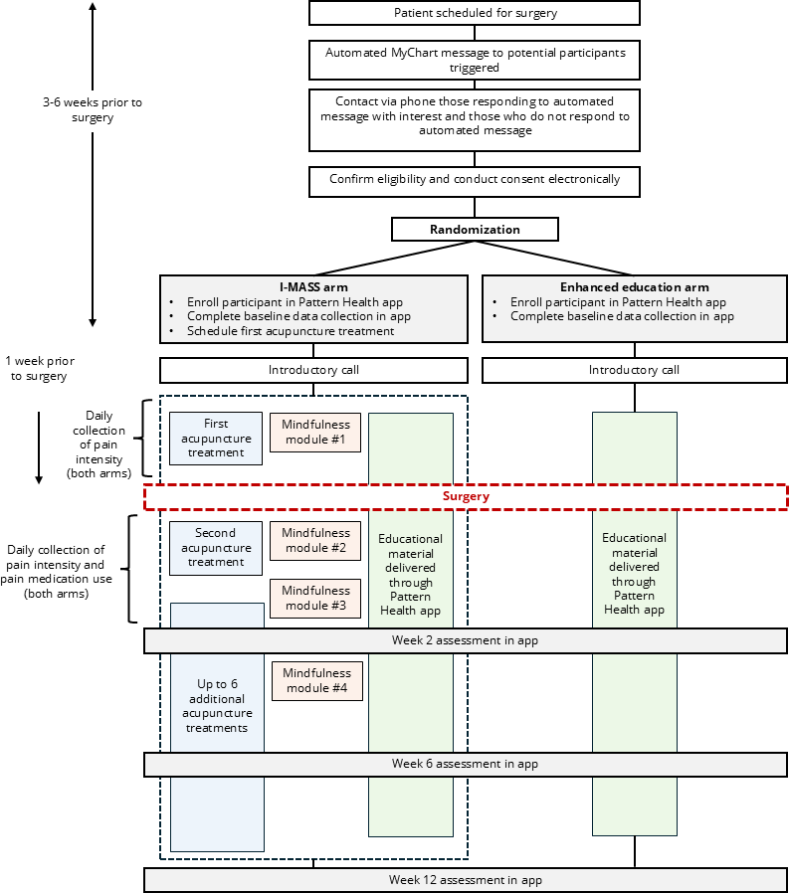
Participant flow through the Integrating Mindfulness and Acupuncture after Spine Surgery (I-MASS) trial.

For all 3 recruitment strategies, calls to potential participants will include an explanation of the purpose of the study; what will be done during the study and length of participation; potential risks and benefits of participating; and verification that the participant has the right to leave the study at any time. Once informed, if the patient is willing to participate in the study, they will be sent a link to complete the eConsent via REDCap. The process for recruitment and enrollment is depicted in Figure 1.

### Allocation

The primary study biostatistician developed a 1:1 randomization schedule using a random number generator. Because this is a planning study, the principal investigators, research coordinators, and clinicians (ie, acupuncturists and orthopedic surgery teams) will not be blinded to the randomization. One of the 2 biostatisticians will be blinded. A study coordinator will randomize consented participants and inform them of their randomization group via telephone. During this call, the coordinator will schedule the participant’s first acupuncture appointment (I-MASS plus education arm only) and send the participant a link to download the Pattern Health mobile app (both arms). Upon randomization, the participants’ demographic details, contact information, and surgery date will be entered into the Pattern Health dashboard before the link is sent, allowing us to designate their allocation and start date in the app. The participant will be reminded to complete baseline surveys. They will also be reminded that a study team member will contact them approximately 1 week prior to their surgery (described in the “Introductory Call” section) to review their preoperative study tasks.

### Study Intervention

#### Overview

Participants will be randomized to one of two study arms: (1) mindfulness and acupuncture (ie, the I-MASS program) plus enhanced education or (2) enhanced education only (Figure 1).

#### Mindfulness

Participants will complete 4 week-long mindfulness modules using the Pattern Health mobile app (1 wk prior to surgery, 3 wk after surgery). Each module begins with a short (4‐5 min) background video describing the mindfulness technique for that week. Participants then complete daily guided practice sessions (6‐8 min each) that focus on that week’s mindfulness technique (Table 1) [45,46]. Each module builds upon skills learned in the previous module. The preoperative module (module 1) discusses the rationale for incorporating mindfulness into the recovery process and teaches how to develop an awareness of breath. Awareness of breathing is a fundamental mindfulness technique that begins to cultivate skills of mindful nonreactive observation. Module 2 begins 2 days after surgery and focuses on building awareness of body systems. This module cultivates skills of observing, describing, and nonjudgmental attention. Module 3 focuses on awareness of emotion and mindful acceptance. This module aids in acknowledging difficult emotions and cultivating feelings of kindness and compassion towards oneself and others. The fourth and final module introduces awareness of the senses. It is designed to further build attention, concentration, observation, nonjudgment, and nonreactivity skills. The modules present mindfulness as a learned skill that participants are encouraged to continue using after completing the modules.

**Table 1.** Content of the mindfulness modules delivered through the Pattern Health app.

Module	Content	Purpose
Week 1	Rationale for mindfulness Learn to use awareness of breathing	Begin to cultivate skills of mindful nonreactive observation
Week 2	Build awareness of body systems that are working well or less well	Continue to cultivate skills of observing, describing, and nonjudgmental attention
Week 3	Practice awareness of emotion and mindful acceptance	Aids in acknowledging difficult emotions and cultivating feelings of kindness and compassion towards oneself and others
Week 4	Introduce awareness of the senses	Build the skills of attention, concentration, observation, nonjudgment, and nonreactivity

Automated reminders in the app will alert the participant to complete each daily practice session. Participants can choose when they receive these push notifications. In the first week’s introductory mindfulness video, they are encouraged to complete mindfulness sessions while in a comfortable location and during a time when they will not be interrupted. All participant activity in the app, including access to module content, can be tracked in the Pattern Health dashboard.

#### Acupuncture

Participants will receive up to 8 total acupuncture treatments at participating outpatient clinics. The initial acupuncture treatment will be scheduled to occur the week prior to surgery. The second acupuncture treatment will be planned to occur within 10 days of surgery, as soon as the participant can safely travel. Participants will have up to 12 weeks after surgery to complete their acupuncture treatments. We will take a pragmatic approach to the timing and cadence of acupuncture follow-up visits, aiming for visits to occur every 5 to 10 days at the discretion of the participant and their acupuncturist. Approximately every 2 weeks after surgery, participants are sent an automated reminder in the mobile app to make sure their next acupuncture treatment is scheduled.

Acupuncturists will also undergo a one-hour training session designed by the study psychologist to facilitate discussion about mindful awareness and nonjudgmental thoughts regarding pain, allowing participants to reflect on their experiences during the acupuncture session. We will not mandate a specific acupuncture protocol. Both body and auricular acupuncture techniques can be used during treatments. Because acupuncture is commonly delivered to support whole-person health, acupuncturists may target other needs (eg, reduce inflammation and aid in digestion) through treatment in support of postoperative recovery. Specific components of each acupuncture treatment will be recorded according to the revised STRICTA (Standards for Reporting Interventions in Clinical Trials of Acupuncture (Checklist 1) [47] to allow for post hoc assessment of treatment fidelity. Detailed acupuncture note templates will be developed with input and feedback from study acupuncturists, with the level of detail consistent with that needed to appropriately report the intervention content using STRICTA guidelines. All acupuncture charting will be done in study-specific REDCap forms. All acupuncturists involved in the study will undergo a screening process conducted by study personnel to ensure they hold valid licensure, maintain appropriate insurance coverage, and have completed relevant education and training. The study does not mandate a minimum number of years in practice.

#### Enhanced Education

Enhanced education consists of informational materials specially curated and delivered in the Pattern Health mobile app during the appropriate phase of recovery (Table 2). All educational content is from the Healthwise.net Duke Health Library. Educational topics include learning how to lift and sit when experiencing pain, proper precautions following surgery, how to ease back into daily activities following surgery, and ways to self-manage pain. Education is in the form of short reading materials or videos. Participants in both study arms will receive the same enhanced education, which starts in the preoperative week and continues for 6 weeks following surgery. Automated reminders in the app will alert the participant when new educational material is available. Once educational materials are first accessed, they remain available to participants in the app throughout the course of the trial.

**Table 2.** Educational content delivered in both trial arms.

Timing of delivery^a^	Content
The week prior to surgery	Spine pain Heat or ice for spine pain Proper lifting Stress and spine pain Precautions after spine surgery
First week after surgery	Getting in and out of bed Self-massage Spine pain: keeping it from coming back Proper sitting for a healthy spine
Second week after surgery	Easing back into your daily activities
Third week after surgery	Getting support when you have spine pain Spine pain and sex Returning to work with spine pain
Sixth week after surgery	Exercises to reduce pain Keep moving

a After participants access materials for the first time, the content is made available in an education library within the app for the remainder of their time in the trial (through 12 wk after surgery).

#### Introductory Call

All participants will receive a telephone call from a study team member during the week before surgery. For participants in both study arms, the study team member will reinforce the importance of the enhanced education content, answer any questions about the program, remind participants of when questionnaires will be administered through the mobile app, and troubleshoot challenges with accessing or navigating the app. For participants in the I-MASS arm, the study team member will also reinforce mindfulness best practices (eg, find a comfortable place to practice, minimize distractions), review the benefits of combining mindfulness and acupuncture, and review how and when to schedule acupuncture treatments.

#### Other Treatments

Trial participation will not preclude the participant from receiving any other care deemed necessary by their health care team and surgeon, including the use of pain medication.

### Assessments

#### Overview

All assessments presented below, except those indicated otherwise, will be collected at baseline (preintervention) and at 2 weeks, 6 weeks, and 12 weeks after surgery (Table 3). Sociodemographic and health history data will be collected through REDCap. All other self-reported measures will be collected in the Pattern Health app. The app will send an automated notification to the participant when a new assessment is due.

**Table 3.** Primary and secondary outcome measures collected in Integrating Mindfulness and Acupuncture after Spine Surgery (I-MASS).

	Measure description	Time frame
Primary measures		
CSQ-8^a^	CSQ-8 assesses credibility and satisfaction with health-related services (8 items, 4-point Likert, with higher scores indicating higher credibility or satisfaction).	12 weeks after surgery
Recruitment	Percentage of contacted patients who are eligible and agree to participate	Baseline
Data collection	Percentage of participants who complete self-report follow-up questionnaires at each of the 4 timepoints.	Up to 12 weeks after surgery
Number of completed acupuncture visits	Percentage of acupuncture treatments completed out of 8 possible treatments	Up to 12 weeks after surgery
Number of completed mindfulness modules	Percentage of completed mindfulness modules out of 4 possible in the mobile app	Up to 12 weeks after surgery
Secondary measures		
PROMIS-29^b^ version 2 0	This profile assesses pain intensity using a single 0‐10 numeric rating item and 7 health domains (physical function, fatigue, pain interference, depressive symptoms, anxiety, ability to participate in social roles and activities, and sleep disturbance) using 4 items per domain and a 1-item measure of pain intensity.	Baseline (prior to surgery), 2 weeks, 6 weeks, and 12 weeks after surgery
Daily pain intensity	Daily pain intensity during the week prior to surgery and for 2 weeks following surgery will be measured with the following two questions: (1) please rate your worst pain in the last 24 hours on a scale from 0=no pain to 10=worst possible pain, and (2) please rate your average pain in the last 24 hours on a scale from 0=no pain to 10=worst possible pain.	Daily the week before surgery and for 2 weeks after surgery
SPARE-FA^c^ Short form	The SPARE-FA short form is a 4-item questionnaire that measures characteristics of pain-related fear avoidance, catastrophizing, and kinesiophobia. Higher scores indicate higher levels of fear avoidance.	Baseline (prior to surgery), 2 weeks, 6 weeks, and 12 weeks after surgery
Medication Prescriptions	We will collect self-reported information on medication prescriptions for pain (eg, opioids, benzodiazepines, gabapentin or neurontin, and NSAIDs^d^) at baseline, 6 weeks, and 3 months. We will also collect self-reported pain medication use daily during the first 2 weeks following surgery.	Baseline (prior to surgery), 2 weeks, 6 weeks, 12 weeks after surgery
Number of hospital readmissions related to spine surgery	Identified through ADT^e^ alerts in the Epic EHR^f^ and periodic chart reviews through 12 weeks.	Up to 12 weeks after surgery
Number of emergency room visits related to spine	Identified through ADT alerts in the Epic EHR and periodic chart reviews through 12 weeks.	Up to 12 weeks after surgery
Adverse events	Identified through ADT alerts in the Epic EHR and periodic chart reviews through 12 weeks. Adverse events are also documented in acupuncture treatment notes.	Up to 12 weeks after surgery

a CSQ-8: 8-item Client Satisfaction Questionnaire.

b PROMIS-29: 29-item Patient-Reported Outcomes Measurement Information System.

c SPARE-FA: Screening for Pain Vulnerability and Resilience–Fear Avoidance.

d NSAID: nonsteroidal anti-inflammatory.

e ADT: Acceptance Determination Tool.

f EHR: electronic health record.

#### Sociodemographic and Health History Information

A baseline questionnaire will be administered via REDCap. We will collect data regarding age, sex at birth, gender, race, ethnicity, education, employment status, annual household income, marital status, and disability insurance status. We will also collect zip codes, spinal surgical history (has the participant had prior spinal surgery and if so, list the dates), self-reported existing or comorbid medical diagnoses using the Charlson Comorbidity Index [48], self-reported overall current health status rated on a 5-point Likert scale from poor to excellent, the revised graded chronic pain scale [49], and previous acupuncture and mindfulness use (“Have you ever used these treatments in the past?”).

#### Primary Outcomes

Consistent with the NIH stage model and feasibility and acceptability study guidelines, our primary outcomes are the following measures of feasibility, acceptability, appropriateness, satisfaction, and fidelity:

1. Feasibility of recruitment: we will calculate the percentage of contacted participants who were eligible for the study and who agreed to participate.
2. Feasibility of data collection: we will calculate the percentage of participants who complete the self-report questionnaires at 4 time points: baseline, 2 weeks, 6 weeks, and 12 weeks. We will also calculate the percentage of participants who complete the daily pain intensity and medication use questionnaires (described in the “Secondary Outcomes” section) on more than half the days in postoperative weeks 1 and 2.
3. Treatment satisfaction: we will assess satisfaction with the I-MASS program using the modified Client Satisfaction Questionnaire-8, an 8-item questionnaire designed to assess satisfaction with health-related services [50]. This measure has been modified to meet the needs of this study and includes questions regarding usability, satisfaction, and acceptability.
4. Number of completed acupuncture treatments: we will calculate the number and percentage of acupuncture treatments completed out of 8 possible treatments.
5. Number of completed mindfulness modules: we will calculate the number and percentage of mindfulness modules completed out of 4 possible modules in the mobile app.

#### Secondary Outcomes

Secondary outcomes included the following patient-reported outcomes, as well as health care use, including medication use, emergency department visits, and hospital readmissions:

1. 29-item Patient-Reported Outcomes Measurement Information System, version 2.0*:* this 29-item self-report questionnaire assesses pain intensity using a single 0‐10 numeric rating item and 7 health domains (physical function, fatigue, pain interference, depressive symptoms, anxiety, the ability to participate in social roles and activities, and sleep disturbance) using 4 items per domain and 1 item for pain intensity [51]. Higher scores indicate more of the item being measured, which is positive for physical function and participation in social roles, and negative for all other scores.
2. Daily pain intensity: the following questions will be asked of patients daily during the week prior to surgery and in the 2 weeks following surgery: please rate your worst pain in the last 24 hours on a scale from 0=no pain to 10=worst possible pain, and please rate your average pain in the last 24 hours on a scale from 0=no pain to 10=worst possible pain [52]. Daily pain intensity will be collected in the 2 weeks following surgery because intensity and variability of pain during this timeframe are known to predict the persistence of postoperative pain [53]. Using multiple, repeated pain ratings provides a more ecologically valid and comprehensive assessment of the pain experience than single measures [54]. This method will also allow us to assess the feasibility and completeness of daily pain measure collection using the app, which will inform our decision to use this method in a future trial.
3. Screening for Pain Vulnerability and Resilience–Fear Avoidance Short-Form: this is a 4-item questionnaire that measures characteristics of pain-related fear avoidance, catastrophizing, and kinesiophobia [55]. Higher scores indicate worse outcomes.
4. Medication prescriptions: medication use for pain will be collected by self-report questionnaires. Medication prescriptions will also be extracted through an Epic chart review, and harmonized NIH Pain Management Pragmatic and Implementation Studies for the Management Initiative definitions [56] will be used to identify prescribed opioid medications. A standard formula from the Centers for Disease Control and Prevention Morphine Equivalent Factors will be used to compare opioid doses across classes. Medication use will be collected at baseline (before intervention) and daily during the 2 weeks following surgery. For each daily questionnaire, we will collect information on the type or name of medication, the number of pills taken in the last 24 hours, and the number of milligrams of each pill taken.
5. Number of hospital readmissions related to spine surgery from enrollment to study termination: collected through a Duke Acceptance Determination Tool alerts in the Epic EHR and periodic chart reviews through 12 weeks.
6. Number of emergency room visits related to spine surgery from enrollment to study termination: collected through Acceptance Determination Tool alerts in the Epic EHR and periodic chart reviews through 12 weeks.

#### Adverse Events

We will count the number of adverse events related to program participation among all enrolled participants, and the raw number of events will be reported by treatment arm.

### Sample Size

We will enroll 50 participants (25/50, 50% per arm), which is suitable to achieve our feasibility and acceptability aims, including determining whether attrition rates will make a larger trial feasible. This approach and sample size are consistent with similar successful trial planning studies conducted on digital health and integrative medicine interventions, including multicomponent intervention studies [46,57-61]. Consistent with the design of our funding mechanism, this trial is neither powered for efficacy nor aimed to provide such information.

### Statistical Methods

Demographic data will be reported as frequency and percentage of nonmissing values for categorical characteristics. Continuous demographic variables will be summarized as mean, SD, median, IQR, and range. We will enumerate the number of participants who meet the “Yes” definitions for all binary feasibility and acceptability outcomes. We will report the proportion of patients that met the definition at each time point with 95% CIs and descriptively compare these figures to benchmark values. For all secondary outcomes, we will report summary statistics (mean, SD for normal distributions; median, IQR for skewed outcomes) of all measures at all time points in which they are collected. This will be done overall and by treatment arm. Since we will not perform tests of efficacy and the rate of data missingness is a primary outcome, we will not have the need to use any techniques to account for missing data (eg, multiple imputation).

We set the following feasibility and acceptability benchmark values for primary outcomes based on similar pilot studies of technology-enabled behavioral interventions for musculoskeletal populations [46,57-61]:

- Feasibility of recruitment: overall, ≥60% of eligible and contacted patients will agree to participate
- Feasibility of data collection: among enrolled participants, ≥70% will complete self-report questionnaires at 4 time points: baselines, 2 weeks, 6 weeks, and 12 weeks.
- Treatment satisfaction: among enrolled participants, ≥70% will endorse a score higher than the midpoint on each of the 6 questions of the modified at 12 weeks. This will be a binary measure of “Yes” (scored a 3 or 4) or “No” (scored a 1 or 2).
- Feasibility of acupuncture: among enrolled participants, ≥70% will complete at least 4 acupuncture treatments out of 8 possible treatments.
- Feasibility of mindfulness: among enrolled participants, ≥70% will complete at least 3 mindfulness modules out of 4 possible modules.

### Dissemination Plans

The results of our study will be presented at scientific conferences and manuscripts in the topical areas of orthopedics, pain management, and complementary and integrative health.

### Ethical Considerations

The trial will be conducted in accordance with Good Clinical Practice guidelines. The trial was registered at ClinicalTrials.gov (NCT06429072), and the investigational plan was approved by the DUHS IRB on May 14, 2024 (approval: Pro00114814). All participants will sign the informed consent before joining the trial. Participation is voluntary, and participants may withdraw at any time without penalty or impact on their access to health care services. Participant data collected during the study will be entered into and stored in a secure REDCap database with access limited to authorized study personnel. Demographic, health-related, and health care use data extracted from the electronic health record will be transferred via Secure File Transfer Protocol portal managed by DUHS. Only authorized staff will have access to the data. Data files are immediately retrieved and subsequently managed within the internal secure computing environment. Only the minimal required information will be maintained in the dataset after project completion, but all identifiers will be destroyed once the IRB protocol is closed. All data collected through the Pattern Health app will be transferred via Secure File Transfer Protocol portal managed by Duke University. Pattern Health will populate the study-specific identification number into the patient-level participant Pattern Health data extract before providing it to the study investigators at Duke. Duke investigators will rely upon the study-specific identification number to integrate the participant Pattern Health data extract and the electronic health record REDCap dataset for reconciliation, reporting, and analysis. Only named members of the research team will have access to identifiable data. No personal identifying information will be included in the analytic extract. Only deidentified data will be used in publications and presentations. Nominal incentives will be provided for completing questionnaires at each time point: baseline, 2 weeks, 6 weeks, and 3 months. The total compensation will be up to $120 for completing all data collection in the study. This protocol has been prepared in accordance with the CONSORT (Consolidated Standards of Reporting Trials) guidelines Checklist 2.

## Results

Trial enrollment began in August 2024. As of May 9, 2025, 35 participants have been enrolled. Enrollment is expected to be completed in the fall of 2025. Data analysis has not yet been performed. Results are expected to be published in the first half of 2026.

## Discussion

### Principal Findings

We anticipate that the I-MASS program will be acceptable and feasible for patients undergoing spine surgery. I-MASS is highly responsive to the need for mind and body treatments with strong potential to improve recovery and reduce risk for chronic pain and opioid use after spine surgery. Existing treatments have not adequately addressed this need. I-MASS takes a unique approach by using multicomponent treatments with distinct yet complementary therapeutic mechanisms. While acupuncture and mindfulness are commonly studied interventions, to our knowledge, no studies have fully integrated the two as a method of managing pain and improving recovery after spine surgery. Prior studies have found modest effects of acupuncture alone on pain intensity following spine surgery [62]; however, most of these studies have not assessed longer term results or outcomes like pain interference, pain-related disability, or changes in psychological distress. Likewise, mindfulness interventions have shown promising results after spine surgery [63-65], although many studies are limited by small sample size, and none to our knowledge use an asynchronous, entirely digital, self-directed mindfulness program delivered before and after surgery.

The I-MASS program is innovative because it is not simply 2 treatments provided concurrently, but rather an integrated program that includes both synchronous and asynchronous components. Using a mobile app for the delivery of mindfulness has great potential to make nonpharmacologic pain care more efficient and accessible to patients undergoing spine surgery. Importantly, modules teach mindfulness as a self-management strategy that participants can continue using after completing the study. Another important attribute of the program is that delivery of acupuncture is not confined to the academic medical setting. For this trial, we have developed a network of study-approved acupuncture clinics within a broad geographic region. The network allows us to enroll participants who receive surgery at DUHS but do not live in the immediate vicinity of a DUHS clinic. This is important because pain and fatigue that are common prior to and after surgery can limit the ability to travel long distances for treatment. We also take a pragmatic approach to the delivery of acupuncture where treatments are tailored to the unique needs of each participant with the intention of optimizing their postsurgical recovery. This approach closely mirrors usual care. The use of a network of I-MASS–trained acupuncturists and flexibility in treatment delivery are likely to enhance implementation potential for future pragmatic studies.

### Limitations

This study has several limitations. First, we intentionally limited eligibility to patients undergoing single-level procedures of the spine. We did so to reduce the potential for confounding variables that could accompany larger and more complex procedures, like high complication rates. The approach is common for preliminary testing in feasibility and acceptability studies but could limit generalizability to patients with more complex procedures or clinical presentations. Second, acupuncture is delivered by acupuncturists trained in mindfulness and practicing within our I-MASS network. Because participants would need to receive acupuncture treatments from study-approved acupuncturists, we are limited to recruiting patients within a reasonable travel distance of our acupuncture clinics. This limits the number of people who can participate, and participation would be especially difficult for patients living in regions that do not have acupuncture services. Finally, the I-MASS program may not be appropriate for patients with low technical proficiency. Although we have worked closely with Pattern Health to make the app interface intuitive and easy to navigate, the program may not be accessible to patients who cannot access or use mobile apps.

### Future Directions

The results of this feasibility and acceptability trial will provide the information needed to develop a rigorous pragmatic comparative effectiveness study of the I-MASS program for people undergoing spine surgery. If effective, this program has the potential to revolutionize spine care by providing a safe and effective alternative to reliance on medication, including opioids, for postoperative recovery.

### Conclusions

This research aims to provide evidence for the feasibility and acceptability of integrating two effective integrative health interventions, mindfulness and acupuncture, to improve outcomes following spine surgery. The findings will not only contribute to the development of a future, fully powered pragmatic comparative effectiveness trial but also provide insight into the best methods for delivering this integrated program in real-world settings.

## Supplementary material

10.2196/77236 Checklist 1 STRICTA checklist.

10.2196/77236 Checklist 2 CONSORT checklist.

## Abbreviations

DUHS: Duke University Health System
EHR: electronic health record
I-MASS: Integrating Mindfulness and Acupuncture after Spine Surgery
IMC: independent monitoring committee
IRB: institutional review board
NIH: National Institutes of Health
